# Efficacy of Platelet-Rich Plasma Injection in the Management of Lateral Epicondylitis: A Retrospective Assessment

**DOI:** 10.7759/cureus.77267

**Published:** 2025-01-11

**Authors:** Othman H Alzahrani, Gaied Alenezi, Mazen Alsagri, Omar H Alzahrani, Saud Alhussain, Jana M Alfayyadh, Ahmed Alzahrani, Waleed Alrashidi, Ismail H Almogbil, Abdulaziz Altasan

**Affiliations:** 1 Radiology, King Fahad Specialist Hospital, Buraydah, SAU; 2 Medicine, Imam Mohammad Ibn Saud Islamic University, Riyadh, SAU; 3 Medicine, Unaizah College of Medicine and Medical Sciences, Unaizah, SAU; 4 Medicine and Surgery, Almaarefa University, Diriyah, SAU; 5 Orthopedic Surgery, Qassim University, Buraydah, SAU

**Keywords:** chronic elbow pain (cep), disabilities of the arm shoulder and hand (dash), lateral epicondylitis (le), platelet-rich plasma (prp), visual analog scale (vas)

## Abstract

Objective: This study aimed to evaluate the efficacy of platelet-rich plasma (PRP) injections in managing lateral epicondylitis (LE), focusing on pain relief, functional improvement, side effects, and patient satisfaction.

Methods: A retrospective analysis of 19 patients treated with PRP injections at a tertiary care center was conducted. Data included demographic characteristics, Visual Analog Scale (VAS) pain scores, Disabilities of the Arm, Shoulder, and Hand (DASH) scores, and patient satisfaction. Statistical analyses utilized paired t-tests and one-sample proportion tests. Statistical significance was set at p<0.05.

Results: PRP injections significantly reduced VAS pain scores, with 21.05% (4/19) of patients achieving complete pain relief (VAS=0) and 47.37% (9/19) reducing scores to ≤3 (p=0.027). DASH scores improved substantially, with mean scores decreasing from 87.5% (19/19) (95% CI: 82.3-92.7%) pre-treatment to 18.5% (19/19) (95% CI: 12.7-24.3%) post-treatment (p<0.001). Prior corticosteroid use was associated with poorer outcomes (p=0.012). Patient satisfaction was high, with 89.5% (16/19) of patients reporting being "very satisfied" or "satisfied" (p=0.001).

Conclusion: PRP injections offer significant pain relief and functional improvement for patients with LE. This minimally invasive treatment is particularly valuable for high-demand populations, such as professional athletes, due to faster recovery times and reduced need for surgery. However, high patient satisfaction rates underscore its clinical utility. Prospective studies with larger cohorts are needed to further validate these findings and refine PRP protocols.

## Introduction

Lateral epicondylitis (LE) is the most commonly diagnosed condition affecting the elbow, with a prevalence of 1-3% in the general population. While the exact cause is often unclear, it is frequently linked to the repetitive overuse of the wrist extensor or supinator muscles. The extensor carpi radialis brevis (ECRB) is the muscle most frequently involved. Persistent pain, poor functionality, and diminished grip strength are its features, and they can interfere with normal daily tasks and professional performance. It is often seen in middle-aged adults and is linked to repetitive stress from sports like tennis and golf or physically taxing jobs [1,2].

The management of LE encompasses a range of approaches, including rest, nonsteroidal anti-inflammatory drugs (NSAIDs), bracing, physical therapy, extracorporeal shock wave therapy, and botulinum toxin injections. Additional treatments, such as corticosteroid injections (once the standard treatment but now debated), whole blood injections, platelet-rich plasma (PRP) therapy, and various surgical procedures, have also been explored. Corticosteroid injections, widely used since the 1950s, were long regarded as the preferred treatment. However, numerous studies have questioned their long-term effectiveness, prompting the development of alternative biologic injection therapies, including PRP [3,4].

LE is one of the chronic tendinopathies for which PRP therapy has drawn interest as a biologic treatment. PRP has emerged as a widely used cell-free therapy for the treatment of tendinopathy on a global scale. Basic scientific research has consistently demonstrated the positive effects of PRP on tendon health, including enhanced tendon cell proliferation, upregulation of anabolic gene and protein expression, and a reduction in tendon inflammation [5]. Also, concentrated platelets in PRP, which is made from the patient's blood, release growth factors like platelet-derived growth factor (PDGF), vascular endothelial growth factor (VEGF), and transforming growth factor beta (TGF-β), which promote collagen production, reduce inflammation, and help repair damaged tissue [6]. Because of these characteristics, PRP is promising in treating the degenerative alterations which are related to chronic tendinopathies.

Research indicates that PRP provides superior long-term results compared to corticosteroids [7]. For instance, Gosens et al. reported two years of functional improvement and persistent pain alleviation [8]. Also, Thanasas et al. showed the long-term advantages of PRP for chronic elbow tendinopathy [9]. Prior use of corticosteroids has been linked to worse PRP outcomes due to its detrimental effects on tendon structure and healing capacity [10].

PRP injections and surgical intervention yielded comparable pain relief and functional improvement in patients with lateral elbow tendinosis [11]. Consequently, PRP injections could serve as a viable alternative for individuals hesitant about surgery or those who are unsuitable candidates for surgical procedures.

The efficacy of PRP in treating LE at King Fahad Specialist Hospital in Buraydah is assessed retrospectively in this study. It seeks to evaluate functional improvement, pain reduction, and patient satisfaction while determining the variables that influence therapy results.

## Materials and methods

This retrospective study reviewed the medical records of 19 patients treated for LE with PRP at King Fahad Specialist Hospital, Buraydah, between January 2022 and December 2024.

Eligibility criteria included a confirmed diagnosis of LE through clinical and imaging findings (using ultrasound (US) and magnetic resonance imaging (MRI)). To diagnose LE, both US and MRI were utilized. US was employed to evaluate tendon structure and identify abnormalities such as thickening or tears, while MRI was used to provide detailed imaging of soft tissues and surrounding structures, confirming the diagnosis. Inclusion criteria included the availability of complete pre- and post-treatment data and a minimum follow-up period of six months [1,2]. Patients with systemic inflammatory diseases, previous elbow surgeries, or incomplete records were excluded. The study was approved by the hospital's Research Committee on 27-11-2024 (approval number: 607/46/5791) and conducted in accordance with the ethical principles outlined in the Declaration of Helsinki. Also, informed consent for using anonymized data was obtained during treatment.

The PRP injection technique involved collecting 40 mL of blood into a 60 mL syringe containing 6 mL of citrate anticoagulant. The blood was processed in a centrifugation machine at 4500 rpm for six minutes to isolate and concentrate the platelet-rich layer. Typically, 3-4 mL of PRP (L-PRP type) was obtained, and a standardized dose was administered to all patients. No cell counts were performed in this study. The injection was administered using a 23-gauge needle, with a single skin entry made at the most tender point over the lateral epicondyle under ultrasound guidance. The PRP was then injected into the common extensor tendons. Patients were advised to avoid anti-inflammatory drugs and limit activities for four weeks to promote recovery. According to our hospital protocol, patients undergoing PRP therapy receive three PRP injections, administered at intervals of four weeks. This schedule allows sufficient time for the injected platelets to release growth factors, stimulate tissue repair, and promote healing. The approach is designed to maximize therapeutic outcomes while minimizing risks associated with the treatment. The four-week interval is particularly important, as it provides the body with adequate time to respond to each injection, ensuring optimal efficacy across the three sessions.

The collected data encompassed patient demographics, including age and gender, along with clinical outcomes evaluated both prior to and following treatment. Pain intensity was measured using the Visual Analog Scale (VAS), while functional impairments were assessed through the Disabilities of the Arm, Shoulder, and Hand (DASH) score [12]. Additionally, patient satisfaction with the treatment was evaluated during follow-up appointments using a structured questionnaire.

Statistical analysis was performed to compare pre- and post-treatment VAS and DASH scores (paired t-tests) and measure satisfaction rates and relief time (proportion tests). Statistical significance was set at p<0.05.

## Results

The demographic analysis revealed significant patterns in the age and gender distribution of patients with LE treated with PRP therapy. The most represented age group was 36-45 years, accounting for 36.84% (7/19) of participants, with a 95% confidence interval (CI) of 17.1-61.4% (p=0.021). This highlights the prevalence of LE among middle-aged individuals, likely due to repetitive occupational or recreational strain. The second most common age group was 46-60 years, representing 31.58% (6/19) of the cohort, followed by 26-35 years at 21.05% (4/19) and individuals aged above 60 years at 10.53% (2/19). These findings suggest that while LE primarily affects individuals actively engaged in physical activities, it also impacts older adults due to cumulative strain over time. Gender distribution showed that men accounted for 57.89% (11/19) of participants, while women represented 42.11% (8/19). Although men showed a higher proportion, statistical analysis did not reveal a significant gender-based difference (95% CI: 33.5-79.7%; p=0.106) (Table 1).

**Table 1:** Demographic analysis.

Age group (in years)	Gender	Count	Percentage
26-35	Female	2	10.53
	Male	2	10.53
36-45	Female	2	10.53
	Male	5	26.32
46-60	Female	4	21.05
	Male	2	10.53
Above 60	Male	2	10.53
Total		19	100

**Figure 1:**
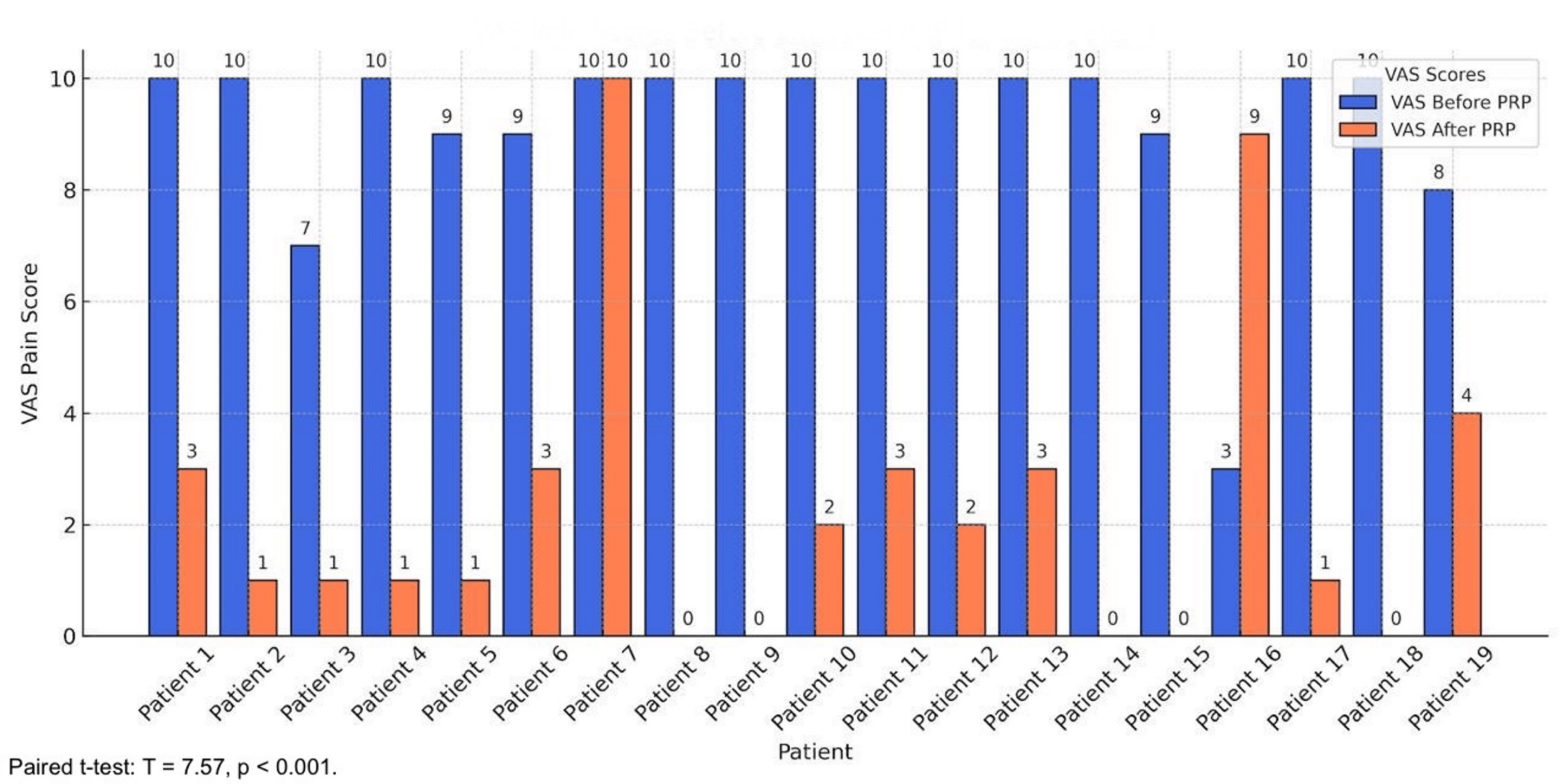
VAS pain scores. The bar chart illustrates that most patients experienced a sharp drop in VAS scores, with some achieving complete or near-complete pain relief after treatment. A paired t-test confirmed this significant reduction in VAS pain scores before and after PRP therapy (T=7.57; p<0.001), demonstrating substantial pain relief across the cohort. VAS: Visual Analog Scale; PRP: platelet-rich plasma

The analysis of VAS pain scores revealed significant reductions, indicating substantial pain relief after PRP therapy. Among the 19 participants, 21.05% (4/19) achieved complete pain relief, transitioning from a pre-treatment VAS score of 10 to a post-treatment score of 0 (95% CI: 8.3-45%; p=0.027). An additional 15.79% (3/19) of patients experienced near-complete pain relief, with scores reducing from 10 to 1, while 5.26% (1/19), who began with a VAS score of 9, improved to a score of 1. Moderate pain relief was observed in 10.53% (2/19) of patients, with scores decreasing from 10 to 2. Overall, 47.37% (9/19) of patients achieved a VAS score of 3 or lower, reflecting clinically meaningful pain reduction across the cohort (paired t-test: T=7.57; p<0.001) (Figure 1).

**Figure 2:**
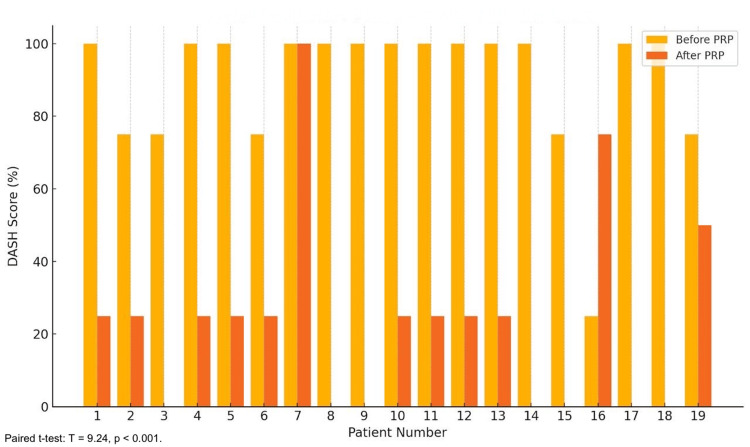
Overall DASH scores. The bar chart highlights a consistent and notable decrease in disability scores for all patients post-treatment. A paired t-test demonstrated a highly significant reduction in overall DASH scores following PRP treatment (T=9.24; p<0.001), reflecting improved physical functionality. DASH: Disabilities of the Arm, Shoulder, and Hand; PRP: platelet-rich plasma

The overall DASH scores demonstrated a statistically significant reduction following PRP treatment, underscoring its effectiveness in alleviating disability and restoring functionality among patients with LE. The mean DASH score decreased from 87.5% (95% CI: 82.3-92.7%) pre-treatment (19 patients) to 18.5% (95% CI: 12.7-24.3%) post-treatment (19 patients) (paired t-test: T=9.24; p<0.001). Patients with higher baseline disability (DASH >90%) accounted for 14/19 patients (73.68%) and experienced the most pronounced improvements, with 13/19 patients (68.42%) achieving post-treatment scores below 20%. Patients with milder pre-treatment scores (DASH <80%) represented 5/19 patients (26.32%) and also demonstrated significant functional gains, emphasizing the broad applicability of PRP therapy in this population. Two patients (2/19, 10.53%) exhibited post-treatment DASH scores exceeding 40%. Both patients had a history of prior corticosteroid use, which may have contributed to structural damage to the tendon itself, thereby compromising the healing process and diminishing the therapeutic response to PRP. These findings reinforce the tolerability and efficacy of PRP as a minimally invasive therapeutic option for reducing disability and improving physical function in patients with LE while also highlighting the potential impact of pre-existing factors, such as corticosteroid use, on treatment outcomes (Figure 2).

**Figure 3:**
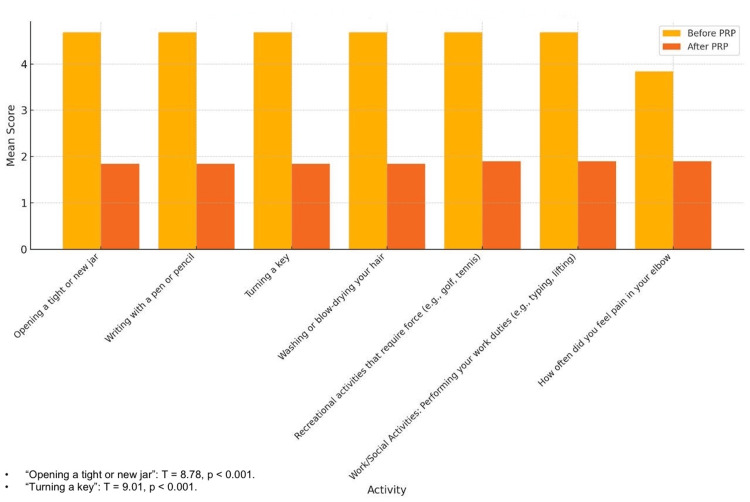
Average activity-specific DASH scores. The figure shows marked reductions in scores across various tasks, emphasizing the restoration of functional abilities essential for daily activities. Significant improvements were observed across all activity-specific DASH scores, with paired t-tests yielding p<0.001 for each task, including "Opening a jar" (T=8.78) and "Turning a key" (T=9.01). DASH: Disabilities of the Arm, Shoulder, and Hand

Activity-specific DASH scores also revealed significant improvements across all functional tasks, indicating a wide-ranging impact of PRP therapy. For example, scores for "Opening a tight or new jar" decreased from 93.6% (4.68/5) (95% CI: 4.5-4.9) before PRP to 36.8% (1.84/5) (95% CI: 1.7-2.0) after PRP (T=8.78; p<0.001). Similarly, "Writing with a pen or pencil" and "Turning a key" showed reductions from 93.6% (4.68/5) and 91.2% (4.56/5) to 36.8% (1.84/5) and 28.4% (1.42/5), respectively (p<0.001 for both). Tasks involving grip strength and force, such as "Recreational activities" and "Performing work duties", also exhibited substantial improvements, with scores dropping from 94.8% (4.74/5) to 40.6% (2.03/5) and from 91.6% (4.58/5) to 29.6% (1.48/5), respectively (p<0.001 for both). These results underscore the effectiveness of PRP in restoring functionality and improving daily task performance for patients with LE (Figure 3).

**Figure 4:**
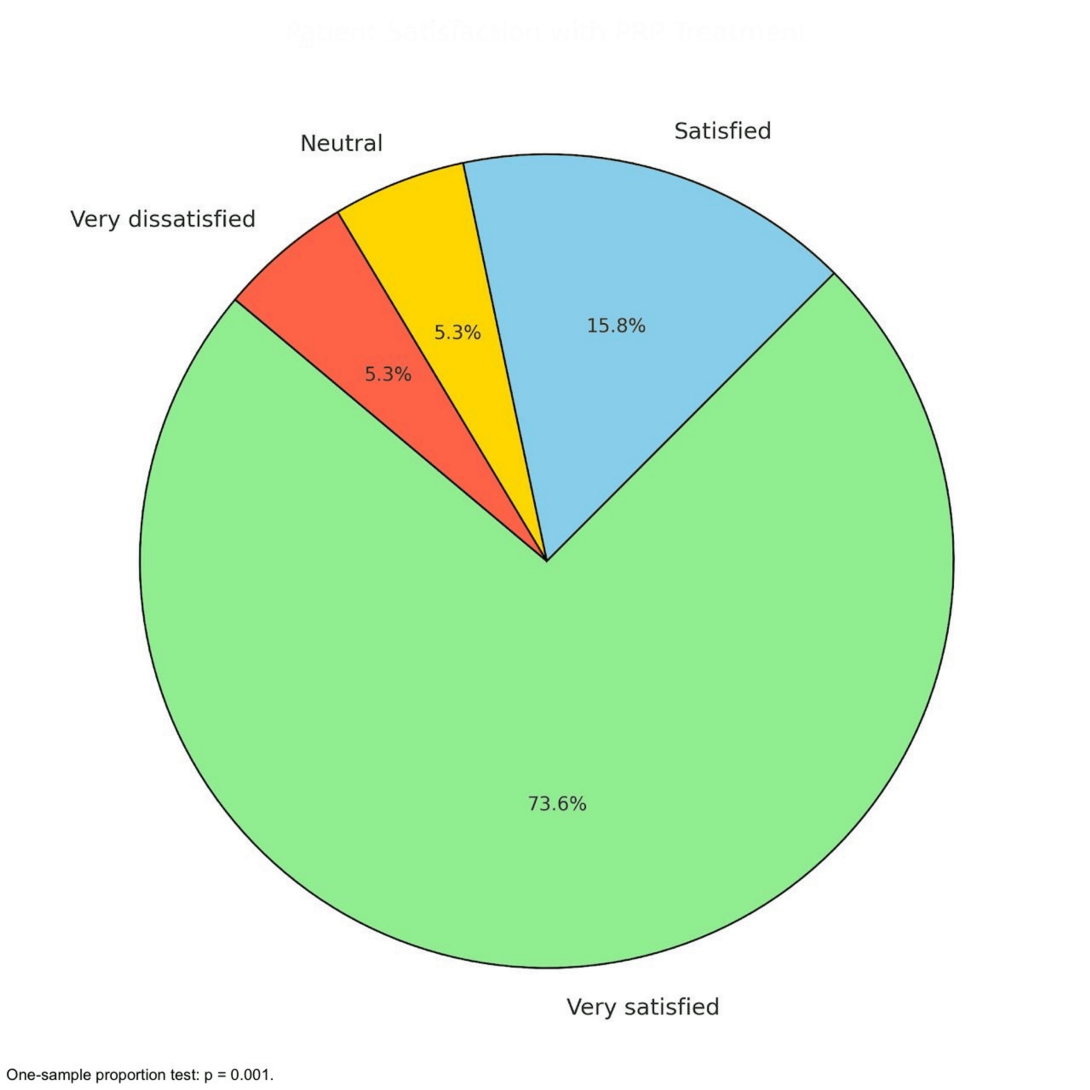
Patient satisfaction. The pie chart demonstrates that a majority of patients reported being very satisfied or satisfied with the treatment, with minimal dissatisfaction. A one-sample proportion test comparing the satisfaction rate (89.5%) (16/19) to a neutral distribution 5.3% (1/19) yielded a statistical significance (p=0.001), underscoring high patient satisfaction with PRP treatment. PRP: platelet-rich plasma

Patient satisfaction with PRP treatment was overwhelmingly positive. Among the participants, 73.7% (13/19) reported being "very satisfied", and 15.8% (3/19) indicated they were "satisfied", yielding a combined satisfaction rate of 89.5% (16/19) (95% CI: 66.9-97.7%; p=0.001). A one-sample proportion test comparing this level of satisfaction to a hypothetical neutral distribution (50%) yielded a statistically significant result, with p=0.001. These results underscore the effectiveness of PRP treatment in enhancing patient satisfaction. Neutral and very dissatisfied responses accounted for 5.3% (1/19) each. These results highlight PRP's success in alleviating pain, restoring functionality, and meeting patient expectations. Collectively, the findings underscore PRP's potential as a minimally invasive, effective, and patient-friendly therapeutic option for LE (Figure 4).

**Figure 5:**
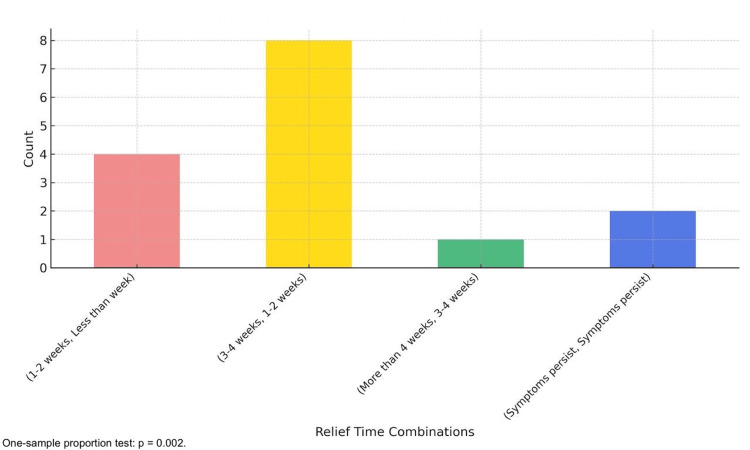
Relief times after the second injection. The bar chart indicates that most patients experienced faster symptom relief after the second injection, with shorter recovery periods being the most frequent outcome. A one-sample proportion test revealed a significant reduction in relief times after the second PRP injection compared to chance (p=0.002), highlighting its cumulative benefits. PRP: platelet-rich plasma

The analysis of relief times following PRP injections revealed significant improvements, particularly after the second injection. Among 15 patients analyzed, 86.67% (13/15) experienced reduced relief times after the second injection (95% CI: 62.12-96.26%; p=0.002). A one-sample proportion test comparing the proportion of improved cases to chance (50%) yielded a statistically significant result, with p=0.002. The most frequent improvement was a transition from relief times of 3-4 weeks after the first injection to 1-2 weeks after the second injection, observed in 53.33% (8/15) of cases. Other patients experienced relief times of less than one week, while 13.33% (2/15) exhibited no improvement, highlighting variability in response to treatment. These findings underscore the cumulative benefits of multiple PRP injections in reducing symptom duration (Figure 5).

## Discussion

The efficacy of PRP injection in managing LE is demonstrated by this retrospective study. The VAS indicates a considerable decrease in discomfort, while the DASH scores show a notable improvement in function. The therapeutic potential of PRP is further supported by the statistically significant improvement and high percentage of positive responses (86.67%). With nearly half reporting VAS scores ≤3 and 21.05% (4/19) of patients experiencing complete pain resolution (VAS=0), PRP therapy was linked to significant pain reduction [13]. The overall mean DASH scores also demonstrated a notable improvement, falling from 87.5% prior to therapy to 18.5% following it, indicating improved functionality and the capacity to carry out everyday tasks [14]. These results are consistent with other research, including that conducted by Gosens et al., which showed that PRP, as opposed to corticosteroid injections, produced long-term benefits in pain and function. The long-term advantages of PRP for treating chronic tendinopathies were also highlighted by Mishra and Pavelko [15].

One noteworthy finding was the correlation between decreased PRP injection efficacy and previous corticosteroid treatments. Patients with a history of corticosteroid usage exhibited higher post-treatment VAS scores. Ben-Nafa and Munro reported significant differences in ultrasonographic outcomes between corticosteroid-treated and PRP-treated groups. In the corticosteroid group, there was a notable reduction in tendon thickness as well as a higher prevalence of cortical erosion. Conversely, the PRP group demonstrated increased tendon thickness and a lower incidence of common extensor tendon tears [16]. These findings highlight the potential regenerative benefits of PRP injection on tendon integrity and healing, positioning it as a promising alternative to corticosteroid injection in the management of tendinopathy. Therefore, clinicians should carefully consider the possible long-term effects of corticosteroid injection.

In a similar vein, Thanasas et al. found that PRP injection was superior to autologous whole blood injections in treating LE. PRP's injection capacity to boost collagen synthesis and encourage tissue remodeling was emphasized by Anitua et al. [17]. PRP's injection potential to treat pain and underlying degenerative changes in chronic musculoskeletal diseases was also highlighted by Andia and Maffulli [18]. PRP injection may lessen or postpone the need for surgery by offering substantial pain relief and functional restoration, especially for high-demand populations or professional athletes during their active season. 

Kim et al. highlighted that PRP injections and surgical interventions yielded comparable results in terms of pain alleviation and functional recovery for patients with lateral elbow tendinosis. These findings suggest that PRP injections can serve as an effective non-surgical alternative, particularly for patients who are apprehensive about surgical procedures or those deemed unsuitable candidates due to underlying health conditions. This equivalence in outcomes reinforces the potential role of PRP as a less invasive treatment option and at least as the first-line treatment in LE.

Overall, patient satisfaction was high, with 89.5% (16/19) reporting they were either "very satisfied" or "satisfied" with their outcomes. These findings align with previous studies, such as those by Gosens et al., which also reported high satisfaction rates and sustained improvements in pain and function following PRP injection. This underscores the tolerability and effectiveness of PRP injection in delivering positive patient-reported outcomes.

This study found significant improvements in symptom relief times with repeated PRP injections, contrasting with Glanzmann and Audigé, who reported no significant differences in outcomes with varying injection numbers. While in our study the patients reported quicker relief and a more significant reduction in pain scores which supports cumulative benefits from multiple PRP injections, Glanzmann and Audigé's findings raise questions about their consistency. Further research is needed to clarify the role of repeated PRP injections in tendinopathy management [19].

In our study, the PRP injection was well-tolerated by all patients, with no significant side effects observed. The only reported adverse effect was immediate post-injection pain, which occurred during the night following the procedure and resolved on average within one day. This suggests that the procedure is generally safe and associated with minimal discomfort, supporting its use as a viable treatment option for LE.

The study presents promising results but has certain limitations. Its small sample size of 19 patients restricts the generalizability of the findings and prevents detailed subgroup analyses. Evaluating the cost-effectiveness of PRP injection in reducing the need for surgical procedures is also essential. Expanding future trials to include a more diverse population with varying activity levels will provide a clearer understanding of PRP's therapeutic potential for LE.

## Conclusions

This study demonstrates the efficacy of PRP injection in managing LE, with significant improvements in pain reduction and functional outcomes. PRP therapy was associated with notable decreases in VAS scores and substantial improvements in DASH scores, highlighting its potential as a non-surgical alternative. Compared to corticosteroid injections, PRP showed superior long-term benefits, including enhanced tendon integrity and reduced structural deterioration, positioning it as a promising first-line treatment for chronic tendinopathies.

The findings also suggest cumulative benefits from repeated PRP injections, though inconsistencies in preparation protocols and small sample sizes limit generalizability. PRP therapy was shown to be as effective as surgical interventions, offering a less invasive option for patients seeking long-term symptom relief and functional restoration. Further research, including large-scale and standardized studies, is needed to optimize PRP protocols, evaluate its cost-effectiveness, and expand its application across diverse patient populations.
